# Multi-omics analysis reveals associations among endophytic microbiome shifts, host transcriptional responses, and metabolic variation across variegated leaf sectors of *Aspidistra elatior*

**DOI:** 10.3389/fpls.2026.1860906

**Published:** 2026-07-08

**Authors:** Yan Wang, Fu Wang, Yan Li, Shanshan Han

**Affiliations:** 1 Shaanxi Institute of Microbiology, Shaanxi Key Laboratory of Qinling Ecological Security, Shaanxi Academy of Sciences, Xi’an, Shaanxi, China; 2 Xi’an Angel Biotechnology Co., Ltd., Xi’an, Shaanxi, China; 3 Bio-Agriculture Institute of Shaanxi Province, Shaanxi Academy of Sciences, Xi’an, Shaanxi, China

**Keywords:** *Aspidistra elatior*, bacterial community imbalance, endophytic bacteria, leaf variegation, metabolomics, plant–microbe association, transcriptomics

## Abstract

Leaf variegation in *Aspidistra elatior* provides a useful system for exploring localized plant–microbiome associations, yet the biological factors linked to sector-specific chlorosis remain unclear. To investigate potential relationships among endophytic microbial communities, host transcriptomic responses, and metabolic variation, we integrated 16S/ITS amplicon sequencing, transcriptomics, and widely targeted LC–MS/MS-based metabolomics across normal green (GG), adjacent green (SG), and chlorotic spot (SS) sectors. SS sectors showed sector-associated bacterial community patterns characterized by lower Shannon diversity relative to SG, enrichment of unclassified *Rickettsiales*, *Stenotrophomonas*, and *Salinivibrio*, and reduced abundance of several *Actinobacteriota*-associated taxa. By contrast, fungal community structure remained comparatively stable across sectors. Transcriptomic analysis identified sector-associated expression differences involving stress- and defense-related genes, including heat shock protein 70 (HSP70) and the F-box regulator SKIP23. Metabolomic profiling identified 52 core differentially accumulated metabolites (DEMs) between SS and SG, of which 37 showed higher abundance in SS. These metabolites included alkaloids, flavonoids, phenolic acids, and amino acids and derivatives. Integrated multi-omics correlation analyses linked host gene-expression modules to metabolite classes and revealed a significant correspondence between bacterial community dissimilarity and metabolic variation across sectors. Together, these findings identify SS sectors as spatially distinct leaf microenvironments in which bacterial community structure, host transcriptional state, and metabolite accumulation vary in parallel, placing localized variegation in a broader microbiome–host–metabolite context.

## 1 Introduction

Plant–microbe interactions represent a rapidly expanding area of research in horticulture and fundamental biology. Among these, the endophytic microbiome that colonizes the interior of plant tissues and acts as the closest interacting component with host cells has emerged as one of the key factors influencing plant physiology, development, and fitness (Partida-Martínez and Heil, 2011). Compared to epiphytes, which are highly susceptible to drastic environmental fluctuations, endophytes can more directly influence plant growth and phenotypic expression through metabolic reprogramming, hormonal regulation, and immune modulation (Yadav, 2025; Wang et al., 2025; Oubohssaine et al., 2026). In ornamental horticulture, leaf variegation, characterized by heterogeneous color patches, serves as a premier model for investigating phenotypic plasticity due to its distinct sector-specific patterning (Ren et al., 2022; Bhardwaj et al., 2025; Han et al., 2026).

The occurrence of leaf variegation has been widely attributed to genetic variation, chloroplast developmental defects, pathogen infection, and environmental stress. Genetically, chimerism arising from mutations in nuclear or cytoplasmic genes is a classical explanation for variegation (Wu et al., 2013). Pathological studies have documented chlorosis induced by various viruses or bacteria (Bertazzon et al., 2017; Sõmera and Sooväli, 2023). Additionally, environmental factors such as light intensity, temperature, and moisture can interfere with pigment metabolism or disrupt chloroplast structure (Yoon and Lang, 1998; Titus and Sullivan, 2001). These mechanisms provide well-established explanations for many variegation phenotypes. At the same time, the high spatial heterogeneity of variegated tissues, including sector-specific anatomical and physiological differences, suggests that local tissue microenvironments may also be associated with phenotypic variation (Olson and Clark, 2021; Dechkrong et al., 2024). In naturally variegated *A. elatior* leaves, visually green and chlorotic sectors often occur in close spatial proximity within the same leaf, providing an opportunity to examine whether endophytic microbial variation is associated with sector-specific host responses. In this context, the endophytic microbiome offers a complementary perspective for understanding localized interactions among host physiology, pigment metabolism, and stress-related responses during leaf variegation.

Within the endophytic microecological network, bacteria and fungi may differ in their sensitivity to host microenvironmental changes. Endophytic bacterial communities are often responsive to host metabolic status and environmental perturbations, and may participate in plant–microbe signaling processes (Tan et al., 2025; Wang et al., 2025; Liu et al., 2026). For instance, *Actinobacteriota-*associated taxa have been reported to contribute to phyllosphere or endosphere stability in some plant systems (Chen et al., 2020; Meng et al., 2025). In contrast, endophytic fungal communities may exhibit relatively stable community structures under certain environmental or host-associated conditions (Kim and Park, 2021; Wang et al., 2025). Additionally, changes in microbial community composition are often accompanied by shifts in host secondary metabolites (*e.g*., alkaloids, flavonoids), suggesting potential bidirectional associations between the microbiome and host metabolism (Tian et al., 2020; Tan et al., 2025). Consistently, Risoli et al. (2025) reported that plant–microbe interaction outcomes in winter wheat under field conditions were associated with complex, cultivar-dependent metabolomic reprogramming involving secondary metabolism and defense-related pathways. However, within the heterogeneous microenvironment of variegated leaves, multi-omics evidence linking sector-specific endophytic microbial variation with host transcriptional and metabolic responses remains limited.

To address this gap, we employed a spatially resolved sector-level multi-omics approach to investigate leaf variegation in the ornamental plant *Aspidistra elatior*. As green and chlorotic tissues occur in close spatial proximity within naturally variegated leaves, this system provides an opportunity to examine localized microbiome–host–metabolome variation under relatively similar plant-background and environmental conditions. By integrating 16S rRNA and ITS amplicon sequencing with transcriptomics and widely targeted LC–MS/MS-based metabolomics, we characterized endophytic bacterial and fungal community structures, host transcriptional profiles, and metabolite accumulation patterns across normal green (GG), adjacent green (SG), and chlorotic spot (SS) sectors. Rather than testing a predefined causal pathway, our study aimed to explore potential associations among sector-specific microbial shifts, host transcriptional responses, and localized metabolic changes during leaf variegation. These findings provide a spatially resolved, hypothesis-generating multi-omics framework for understanding plant–microbiome–metabolite associations in variegated *A. elatior* leaves.

## 2 Materials and methods

### 2.1 Plant materials and sample collection

The wild *A. elatior* plants used in this study were collected from a hillside in Shengli Town, Omei City, Sichuan Province, China (103.499590 E, 29.588910 N, altitude 419 m). Intact and healthy plants were selected in the field and immediately transported to the laboratory. The formal taxonomic identification of the plant materials was conducted by Professor Huyin Cheng (College of Pharmacy, Shaanxi University of Chinese Medicine, Xianyang, China). As *A. elatior* is not classified as an endangered or vulnerable species, no specific permissions or licenses were required for the collection of these wild samples. All field studies and plant collections complied with relevant institutional, national, and international guidelines and legislation, including the IUCN Policy Statement on Research Involving Species at Risk of Extinction and the Convention on International Trade in Endangered Species of Wild Fauna and Flora (CITES). A voucher specimen representing the collected material has been deposited in the Shaanxi Key Laboratory of Qinling Ecological Security under the voucher number SQLES-2024-002.

Fresh leaves without visible external disease lesions, necrosis, insect feeding damage, or mechanical injury were selected for downstream analyses. This criterion was used to exclude obvious external pathological or pest-related damage, but not to assign disease status to any leaf sector. To reduce epiphytic microorganisms, the leaves were first surface-cleaned by immersion in 75% (v/v) ethanol for 2 min, followed by surface sterilization with 5% (w/v) sodium hypochlorite solution for 3 min. The leaves were then rinsed thoroughly three times with sterile water to remove residual disinfectants, and aliquots of the final rinse water were plated on nutrient agar (NA) and potato dextrose agar (PDA). Plates were incubated at 28 °C for 5 days (NA) and 25 °C for 5–7 days (PDA). No visible microbial growth was observed after incubation, confirming effective surface sterilization.

After surface sterilization, GG, SG, and SS tissues were collected from naturally variegated *A. elatior* material within a 10 m × 10 m field sampling area. Because *A. elatior* is a rhizomatous species and shoots within a local patch may be clonally connected, three spatially separated ramets or plant clusters were selected as spatial sampling units, but not as confirmed independent genotypes. From each sampling unit, intact leaves with clearly distinguishable green and chlorotic sectors were collected, and visually distinct leaf regions were dissected using sterile tools. GG samples were obtained from uniformly green leaf regions, SG samples from visually green tissues adjacent to chlorotic spots, and SS samples from chlorotic or pale-yellow spot tissues. These terms were used as neutral phenotypic descriptors and do not imply independently confirmed pathogen-free or diseased status. This sampling scheme generated three spatial subsamples for each sector type, yielding three GG, three SG, and three SS samples that were processed independently for DNA extraction, RNA extraction, and metabolite profiling. All dissected tissues were immediately flash-frozen in liquid nitrogen for 30 s and stored at −80 °C until analysis.

### 2.2 Determination of chlorophyll content

Fresh leaf samples were cut into small pieces, homogenized, and ground under liquid nitrogen. Subsequently, 0.08 g of the powder was weighed and transferred into a PE tube, to which 1 mL of 95% (v/v) ethanol was added, and the mixture was vortexed thoroughly. After centrifugation at 6000 rpm for 2 minutes, the supernatant was collected. This extraction process was repeated three times, and the final volume was brought to 10 mL with 95% (v/v) ethanol. The extraction solvent was used as the blank.

The absorbance of the pigment extract was measured at wavelengths of 663 nm and 646 nm using a 722N spectrophotometer. The chlorophyll a (Chl a) and chlorophyll b (Chl b) contents were calculated as follows:


$$\text{Ca }\left(\text{mg }{\text{L}}^{-1}\right)\;=\;12.72{\text{A}}_{663}\;-\;2.59{\text{A}}_{646}$$

$$\text{Cb }\left(\text{mg }{\text{L}}^{-1}\right)\;=\;22.88{\text{A}}_{646}\;-\;4.67{\text{A}}_{663}$$

$$\text{Ct }\left(\text{mg }{\text{L}}^{-1}\right)\;=\text{ Ca }+\text{ Cb }=\;8.05{\text{A}}_{663}\;+\;20.29{\text{A}}_{646}$$

The chlorophyll content was calculated as:


$$\text{Chlorophyll content }\left(\text{mg }{\text{g}}^{-1}\text{ fresh }\mathrm{weight}\right)\;=\;\text{C}\;\times \text{ V }\times \text{ D}/\left(1,000\;\times \text{ W}\right),$$

where C is the chlorophyll concentration (mg L^-1^), V is the final extract volume (mL), D is the dilution factor, and W is the fresh weight of the sample (g).

### 2.3 DNA extraction and 16S/ITS amplicon sequencing

Total genomic DNA was extracted from the samples using the CTAB method. DNA concentration and purity were assessed using 1% agarose gel electrophoresis. DNA was diluted to 1 ng/μL using sterile water according to its concentration. The fungal ITS1-1F region was amplified using the forward primer ITS1-1F-F (5’-CTTGGTCATTTAGAGGAAGTAA-3’) and the reverse primer ITS1-1F-R (5’-GCTGCGTTCTTCATCGATGC-3’). For bacterial communities, the V5-V7 region of the 16S rRNA gene was amplified using the primers 799F (5’-AACMGGATTAGATACCCKG-3’) and 1193R (5’-ACGTCATCCCCACCTTCC-3’). All PCR reactions were carried out with 15 μL of Phusion**^®^** High-Fidelity PCR Master Mix (New England Biolabs); 2 μM of forward and reverse primers, and about 10 ng template DNA. Thermal cycling consisted of initial denaturation at 98 °C for 1 min, followed by 30 cycles of denaturation at 98 °C for 10 s, annealing at 50 °C for 30 s, and elongation at 72 °C for 30 s, with a final elongation at 72 °C for 5 min.

An equal volume of 1 × loading buffer (containing SYBR Green) was mixed with the PCR products, and electrophoresis was performed on a 2% agarose gel for detection. Qualified PCR products were purified using a Qiagen Gel Extraction Kit (Qiagen, Germany), quantified, and pooled in equimolar amounts. Sequencing libraries were constructed using the TruSeq**^®^** DNA PCR-Free Sample Preparation Kit (Illumina, USA) and quantified using Qubit fluorometry and quantitative PCR. After library quality assessment, the 16S and ITS amplicon libraries were subjected to paired-end sequencing on an Illumina NovaSeq 6000 platform by Wuhan Metware Biotechnology Co., Ltd. (Wuhan, China).

### 2.4 Transcriptome sequencing and analysis

Total RNA was extracted from leaf sector samples using an ethanol precipitation protocol with CTAB-PBIOZOL reagent. The extracted RNA was dissolved in 50 µL of DEPC-treated water. The quality and quantity of the total RNA were assessed using a Qubit fluorometer and the Qsep400 high-throughput Bio-fragment Analyzer. mRNA was purified from high-quality total RNA using oligo(dT) magnetic beads and subsequently fragmented into short fragments in fragmentation buffer. First-strand cDNA was synthesized with random hexamer primers, followed by second-strand cDNA synthesis using a strand-specific library construction method in which dUTP was incorporated in place of dTTP. The cDNA underwent end repair, dA-tailing, adapter ligation, PCR amplification, and library construction. The resulting cDNA libraries were subsequently sequenced on an Illumina NovaSeq 6000 platform.

Raw sequencing reads were processed with fastp to remove low-quality reads and adapter sequences, generating clean reads for downstream analysis. *De novo* transcriptome assembly was performed using Trinity. Assembled transcripts were clustered using Corset to generate non-redundant transcript sets. Coding sequences (CDSs) were predicted from assembled transcripts using TransDecoder, and corresponding amino acid sequences were obtained. Functional annotation was performed by sequence alignment against public databases including KEGG, NR, Swiss-Prot, GO, COG/KOG, and TrEMBL databases using DIAMOND. Protein domain analysis was performed using HMMER against the Pfam database. Transcription factors were predicted using iTAK.

Transcript abundance was quantified using RSEM and expressed as fragments per kilobase of transcript per million mapped fragments (FPKM) for expression visualization and descriptive analyses. Differential expression analysis was performed using DESeq2 (v1.22.2) based on raw count data. P values were adjusted using the Benjamini–Hochberg method. Genes with FDR < 0.05 and |log_2_(fold change)| > 1 were defined as differentially expressed genes (DEGs). GO and KEGG enrichment analyses of DEGs were performed using hypergeometric tests.

### 2.5 Widely targeted LC–MS/MS-based metabolomics

Leaf samples were vacuum freeze-dried using a Scientz-100F lyophilizer and then ground into fine powder using a grinding mill (MM400, Retsch) at 30 Hz for 1.5 min. A total of 50 mg of powder was extracted with 500 μL extraction solvent (methanol: water = 50:50 v/v, containing 0.1% hydrochloric acid). Samples were vortexed for 10 min and sonicated for another 10 min, followed by centrifugation at 12,000 rpm for 3 min at 4 °C. The supernatant was collected, and re-extraction was performed once using the same procedure. Combined extracts were filtered through a 0.22 μm membrane prior to liquid chromatography–tandem mass spectrometry (UPLC–MS/MS) analysis.

Metabolite separation was performed using an ExionLC™ AD UHPLC system (AB Sciex) equipped with an Agilent SB-C18 column (1.8 μm, 2.1 × 100 mm). The mobile phase consisted of solvent A (pure water with 0.1% formic acid) and solvent B (acetonitrile with 0.1% formic acid). The gradient program was as follows: 5% B at 0 min, linear increase to 95% B over 9 min, held for 1 min, followed by re-equilibration to initial conditions within 3 min. The flow rate was 0.35 mL/min, column temperature was 40 °C, and injection volume was 2 μL.

Metabolite detection was performed using an ESI-triple quadrupole-linear ion trap mass spectrometer (QTRAP, AB Sciex) operating in multiple reaction monitoring (MRM) mode. Source parameters were set as follows: source temperature 500 °C; ion spray voltage 5500 V (positive mode) and −4500 V (negative mode); curtain gas 25 psi; gas I 50 psi; gas II 60 psi; collision-activated dissociation set to high. Declustering potential (DP) and collision energy (CE) were optimized for each MRM transition under instrument-specific conditions. Metabolite identification was performed by matching retention time, precursor/fragment ion pairs, and MS/MS fragmentation patterns against an in-house database, and relative quantification was based on MRM peak areas.

Quality-control (QC) samples were prepared by pooling equal aliquots from all sector samples and used to monitor analytical stability. Analytical performance was evaluated using TIC overlap, PCA clustering of QC samples, and coefficient of variation (CV) distribution. A total of 92.68% and 100% of metabolites showed CV values below 0.3 and 0.5, respectively.

### 2.6 Anatomical analysis

Longitudinal leaf samples (including veins) from spotted and green regions were fixed in FAA, washed, and bulk-stained with 1% safranin O for 24 h. Samples were then dehydrated through a graded ethanol series (70% to 100%) and cleared using a xylene-ethanol gradient followed by pure xylene. Tissues were infiltrated and embedded in beeswax-supplemented paraffin (Yonghua Paraffin Co., Ltd., Shanghai, China). Sections were prepared using a rotary microtome (KD-202, Kedi Instruments, Jinhua, China), mounted on glass slides, deparaffinized, and counterstained with fast green FCF. Following final clearing and mounting with neutral balsam, tissue morphology was observed under an optical microscope (CX43, Olympus, Tokyo, Japan) at 400× magnification.

### 2.7 Statistical analysis and data visualization

Raw amplicon sequencing data were demultiplexed according to barcode and primer sequences. Low-quality reads were filtered using fastp, paired-end reads were merged using FLASH, and chimeric sequences were detected and removed using vsearch. High-quality reads were processed into amplicon sequence variants (ASVs) using QIIME2. Representative ASV sequences were generated for downstream analyses. Taxonomic assignment was performed using the SILVA 138.1 database for bacteria and the UNITE database for fungi. Chloroplast- and mitochondria-derived sequences were removed from bacterial datasets following taxonomic annotation. Details of removed reads are provided in **Supplementary Table 1**.

Microbial community analyses were conducted in R (v4.5.1). Alpha-diversity indices were calculated using the vegan package, and group differences among GG, SG, and SS sectors were assessed using one-way ANOVA followed by Tukey’s *post hoc* test. Beta diversity was evaluated using Bray–Curtis dissimilarity, and differences in community structure were tested using PERMANOVA with 9,999 permutations. Differentially abundant taxa were identified using linear discriminant analysis effect size (LEfSe), with an LDA score threshold of **>**4.0.

Metabolomic data were analyzed using a combination of multivariate and univariate statistical approaches. Differentially accumulated metabolites (DEMs) were identified using VIP ≥ 1.0, *P* < 0.05, FDR < 0.05, and |log_2_(fold change)| ≥ 1.0. P values were derived from pairwise comparisons, and multiple testing correction was performed using the Benjamini–Hochberg method. Transcriptome–metabolome associations were assessed using Pearson correlation analysis between DEGs and SS-enriched DEMs, with |r| ≥ 0.85 and P < 0.05 defined as significant.

Cross-omics integration analyses were performed using both correlation- and distance-based approaches. Microbiome–metabolome relationships were evaluated using Mantel tests based on Bray–Curtis (microbiome) and Euclidean (metabolome) distance matrices with 999 permutations. Pairwise bacterial genus–metabolite class associations were assessed using Spearman correlation analysis, and significant associations were defined as |r| ≥ 0.65 and *P* < 0.05; the two strongest associations per genus were retained for network construction.

Data processing and visualizations were conducted in R (v4.5.1) and Python (v3.14.5). R packages included vegan (v2.7-5; Oksanen et al., 2025), ggplot2 (v3.5.2; Wickham, 2016), and patchwork (v1.3.1; Pedersen, 2025), while Python-based analyses were performed using pandas (v2.3.3; McKinney, 2010), matplotlib (v3.10.8; Hunter, 2007), and seaborn (v0.13.2; Waskom, 2021). General statistical analyses were performed using IBM SPSS Statistics 26.0 (IBM Corp., Armonk, NY, USA).

## 3 Results

### 3.1 Phenotypic and physiological variation in A. elatior leaf sectors

**Figure 1:**
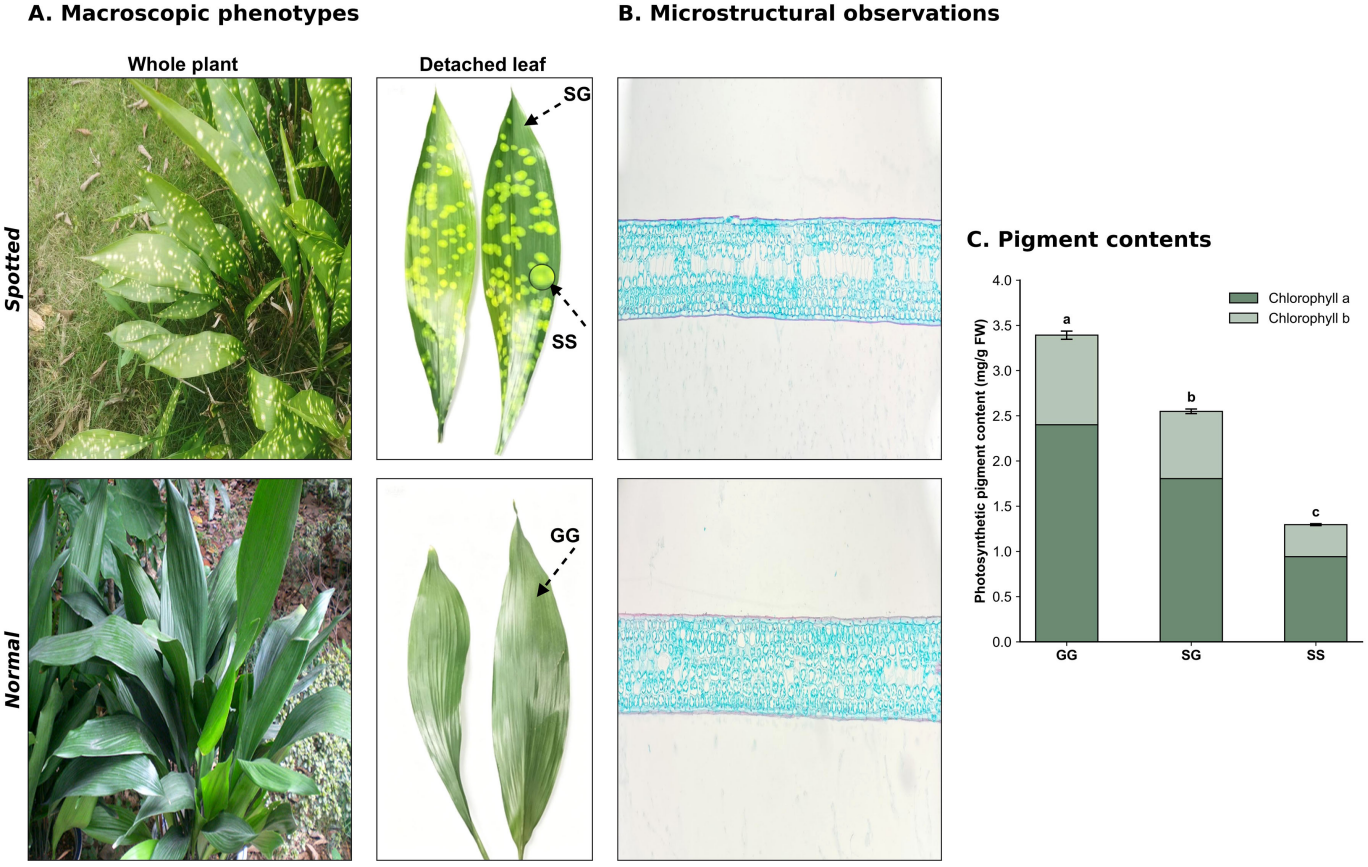
Phenotypic and physiological characteristics of leaf variegation in *A. elatior*. **(A)** Macroscopic phenotypes of GG, SG, and SS sectors; **(B)** microstructural observations of leaf tissues in longitudinal sections; **(C)** Pigment contents in the three sector types. GG, normal green sector; SG, adjacent green sector; SS, chlorotic spot sector.

The leaf surface of naturally variegated *A. elatior* leaves exhibited distinct sector-level phenotypic variation, ranging from uniformly green sectors (GG) to adjacent green sectors (SG) and chlorotic or pale-yellow spot sectors (SS) (**Figure 1A**). Microstructural observations of longitudinal sections (LS) were performed using light microscopy (**Figure 1B**). In GG tissues, the mesophyll cells were densely packed and exhibited well-organized tissue architecture. In SS tissues, the mesophyll layers appeared more disorganized, with increased intercellular spaces and a reduction in pigment-bearing structures, which corresponded to the visible chlorotic regions. Consistent with these anatomical differences, physiological analysis revealed a sector-associated reduction in photosynthetic pigment contents across GG, SG, and SS tissues (**Figure 1C**). In this sector-level comparison, the total chlorophyll content in SG tissues was significantly lower than that in GG tissues (*P* < 0.05), indicating reduced pigment accumulation even in visually green sectors adjacent to chlorotic spots. This reduction was more pronounced in SS tissues, consistent with the visible chlorotic phenotype. Similar sector-associated decreases were observed for both chlorophyll *a* and chlorophyll *b*, supporting the overall reduction in photosynthetic pigment accumulation in SS tissues.

### 3.2 Sector-associated variation in bacterial communities

To examine whether visible leaf-sector phenotypes were associated with variation in bacterial communities recovered from surface-sterilized leaf tissues, we profiled bacterial communities across GG, SG, and SS sectors after computationally removing host chloroplast and mitochondrial sequences. Host-read filtering statistics are provided in **Supplementary Table 1**. Following quality filtering, sequencing depth ranged from 64,996 to 93,203 reads per sample (**Supplementary Table 2**) and rarefaction curves approached saturation for all samples (**Supplementary Figure 1**).

**Figure 2:**
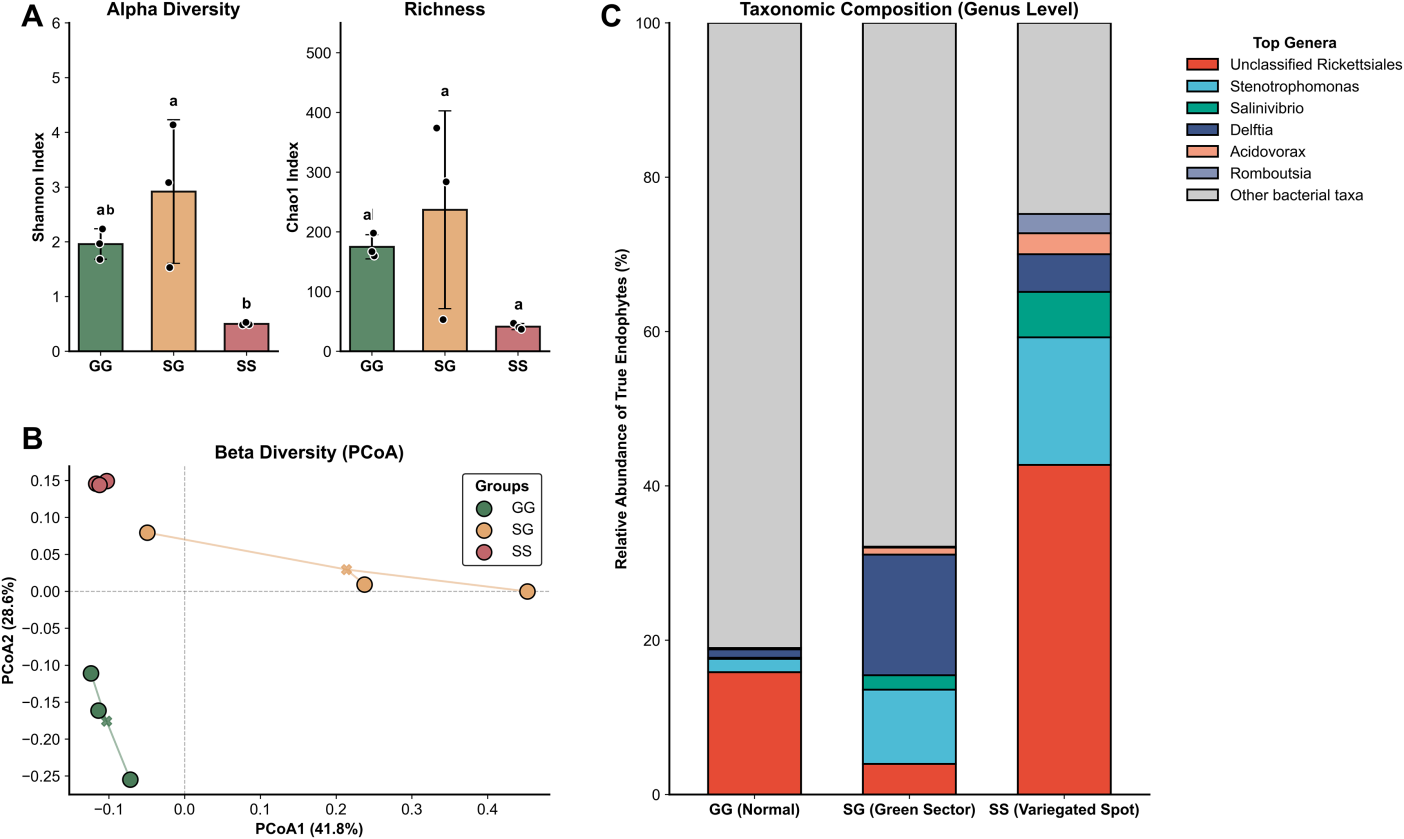
Sector-associated variation in bacterial communities recovered from surface-sterilized leaf tissues of *A. elatior*. **(A)** Alpha diversity of bacterial communities recovered from surface-sterilized leaf tissues across GG, SG, and SS sectors, shown by Shannon index (left) and Chao1 richness (right). Bars represent mean ± SD with individual data points overlaid (n = 3 spatial sector subsamples per sector type). Different lowercase letters indicate significant differences among groups based on Tukey’s multiple comparison test (*P* < 0.05). **(B)** Principal coordinate analysis (PCoA) of bacterial communities based on Bray-Curtis dissimilarity, with spider plot lines connecting individual samples to group centroids. **(C)** Relative abundance of the six most abundant bacterial taxa recovered from surface-sterilized leaf tissues across GG, SG, and SS sectors. Chloroplast and mitochondrial sequences were computationally removed prior to normalization to reduce host-derived sequence interference. Low-abundance taxa were grouped as “Other bacterial taxa”. Sequencing-depth statistics and rarefaction curves are provided in **Supplementary Table 2** and **Supplementary Figure 1**, respectively. GG, normal green sector; SG, adjacent green sector; SS, chlorotic spot sector.

Alpha diversity showed sector-associated variation (**Figure 2A**). GG samples exhibited a Shannon index of 1.96 ± 0.28 and Chao1 richness of 174.9 ± 20.3. SG samples showed greater among-replicate variability, with a Shannon index of 2.92 ± 1.31 and Chao1 richness of 237.0 ± 165.6. In contrast, SS samples displayed the lowest diversity and richness values, with a Shannon index of 0.50 ± 0.02 and Chao1 richness of 41.3 ± 5.5. Tukey’s multiple comparison test identified a significant difference in Shannon diversity between SS and SG samples (*P* < 0.05), whereas Chao1 richness did not differ significantly among the three sector types.

Principal coordinate analysis (PCoA) based on Bray-Curtis dissimilarity showed that GG and SS samples appeared separated in the ordination space, whereas SG samples were more dispersed between the two groups (**Figure 2B**). Pairwise PERMANOVA did not detect significant differences among the three sector comparisons (*P* > 0.05). Nevertheless, the GG–SS comparison explained the largest proportion of variation (R² = 0.885), compared with the GG–SG (R² = 0.466) and SG–SS (R² = 0.457) comparisons.

Taxonomic profiling showed differences in relative abundance patterns among the three sector types (**Figure 2C**). In GG samples, the six most abundant genera were relatively evenly distributed, with unclassified *Rickettsiales* accounting for 16.5% of the total relative abundance. In SG samples, *Delftia* accounted for 17.9% of the total relative abundance. In SS samples, unclassified *Rickettsiales* represented the dominant bacterial group, accounting for 43.1% of the total relative abundance, while *Stenotrophomonas* accounted for 16.0%. The remaining low-abundance taxa were grouped as “Other bacterial taxa”.

### 3.3 Sector-associated enrichment patterns of bacterial taxa

**Figure 3:**
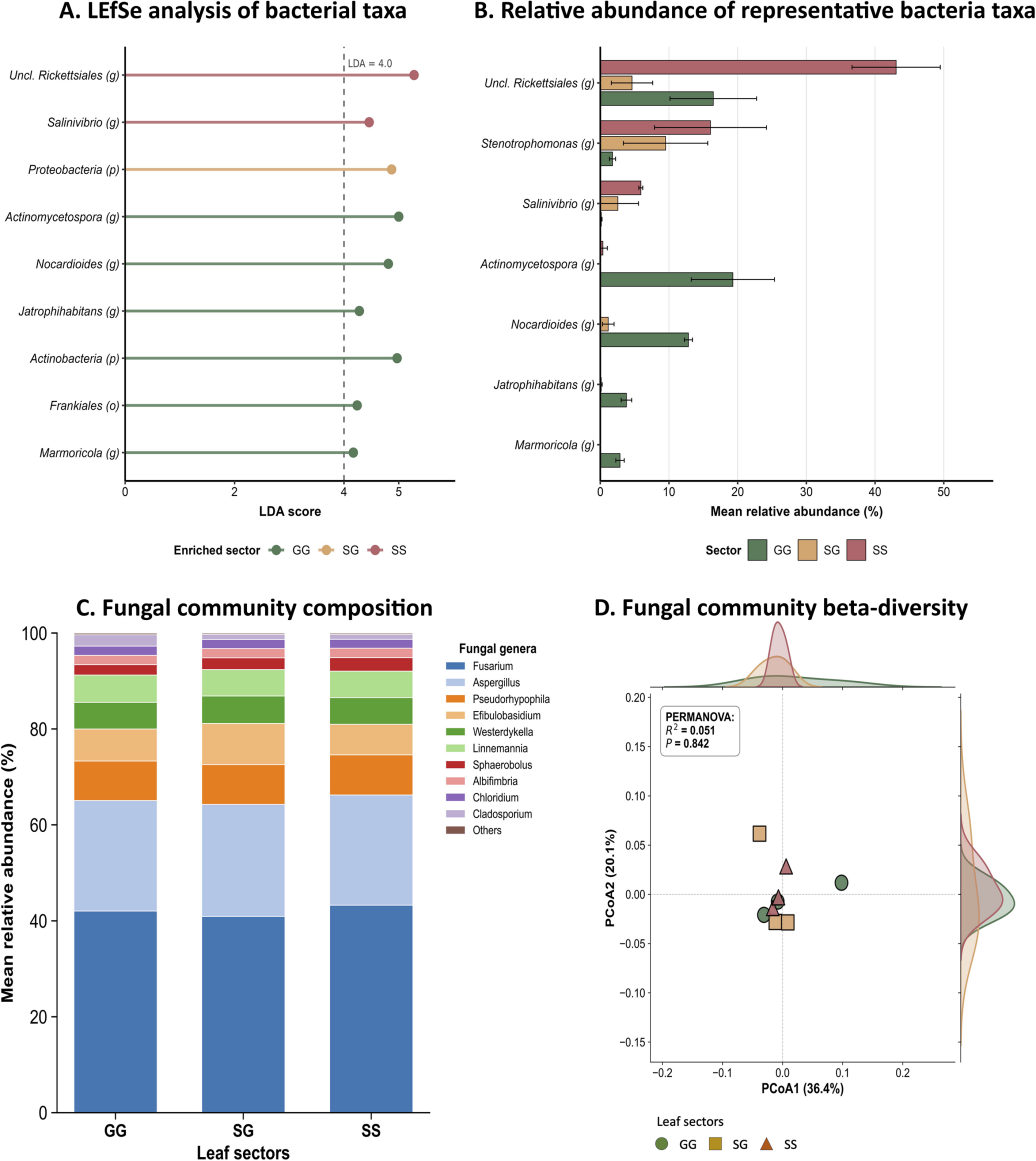
Sector-associated bacterial biomarkers and fungal community patterns across *A. elatior* leaf sectors. **(A)** LEfSe-derived linear discriminant analysis (LDA) scores of bacterial biomarkers associated with GG, SG, and SS sectors. The dashed line indicates the LDA threshold of 4.0. **(B)** Mean relative abundance of representative sector-associated bacterial taxa across GG, SG, and SS sectors. Bars represent mean ± SD calculated from three spatial sector subsamples per sector type. **(C)** Stacked bar plot showing the mean relative abundance of the 10 most abundant fungal genera and other low-abundance genera across GG, SG, and SS sectors. Relative abundance values were averaged from three spatial sector subsamples per sector type. **(D)** Principal coordinate analysis based on Bray–Curtis dissimilarity illustrating fungal community beta-diversity. Marginal plots show the distribution of samples along each coordinate axis. PERMANOVA statistics are shown in the panel. GG, normal green sector; SG, adjacent green sector; SS, chlorotic spot sector. Taxonomic ranks are shown in parentheses: p, phylum; o, order; g, genus. “Uncl.” denotes unclassified taxa.

Using Linear Discriminant Analysis Effect Size (LEfSe, LDA > 4.0), we identified bacterial taxa differentially associated with the three leaf sector types (**Figure 3A**). Within SS sectors, the endophytic community exhibited lower Shannon diversity relative to SG and pronounced taxonomic dominance. As shown in **Figure 3B**, an unclassified *Rickettsiales* taxon was the most abundant bacterial group in SS sectors, reaching a mean relative abundance of 43.1% after computational removal of host chloroplast and mitochondrial sequences. *Stenotrophomonas* and *Salinivibrio* were also enriched in SS sectors, accounting for 16.0% and 5.9% of the total relative abundance, respectively. Collectively, these taxon-level patterns characterized the SS-associated bacterial profile as being dominated by a limited number of taxa.

In contrast, GG sectors harbored an *Actinobacteriota*-enriched community. Sector-associated biomarkers included the genera *Actinomycetospora* (19.3%), *Nocardioides* (12.8%), and *Jatrophihabitans* (3.8%), which were nearly undetectable in SS samples, underscoring their association with the GG sector phenotype.

SG sectors displayed a bacterial community profile intermediate between those of GG and SS sectors. *Proteobacteria*-associated taxa were relatively enriched in SG samples, with abundance levels generally falling between those observed in GG and SS. Overall, SG samples occupied an intermediate position between GG and SS in bacterial community composition.

### 3.4 Comparative stability of endophytic fungal communities across leaf sectors

To investigate whether endophytic fungi were associated with visible leaf-sector phenotype in *A. elatior*, we analyzed fungal community composition and beta-diversity across GG, SG, and SS sectors.

In contrast to the sector-associated taxonomic patterns observed in bacterial communities, the fungal microbiome exhibited comparatively stable compositional patterns across leaf sectors (**Figure 3C**). At the genus level, fungal assemblages were consistently dominated by *Fusarium* and *Aspergillus*, which together accounted for over 50% of total sequences across all samples. Other abundant genera, including *Pseudorhypophila* and *Efibulobasidium*, also maintained consistent relative abundances across GG, SG, and SS samples.

Under the current sampling design and analytical resolution, no fungal genus was found to be significantly enriched or depleted in SS samples compared to GG samples. Beta-diversity analysis further supported the relative stability of the mycobiome. Principal Coordinates Analysis (PCoA) based on Bray-Curtis dissimilarity revealed extensive overlap among GG, SG, and SS samples in the ordination space (**Figure 3D**). PERMANOVA did not detect a significant effect of leaf sector type on fungal community structure (R^2^ = 0.051, *P* = 0.842), with the sector variable explaining only 5.1% of the total variance. These findings suggest that the endophytic fungal community was largely conserved across phenotypically distinct leaf sectors under the current sampling resolution. Compared with the sector-associated variation observed in bacterial taxonomic composition, the fungal community appeared less responsive to leaf-sector phenotype differentiation in the present dataset.

### 3.5 Sector-associated metabolic variation in A. elatior leaves

**Figure 4:**
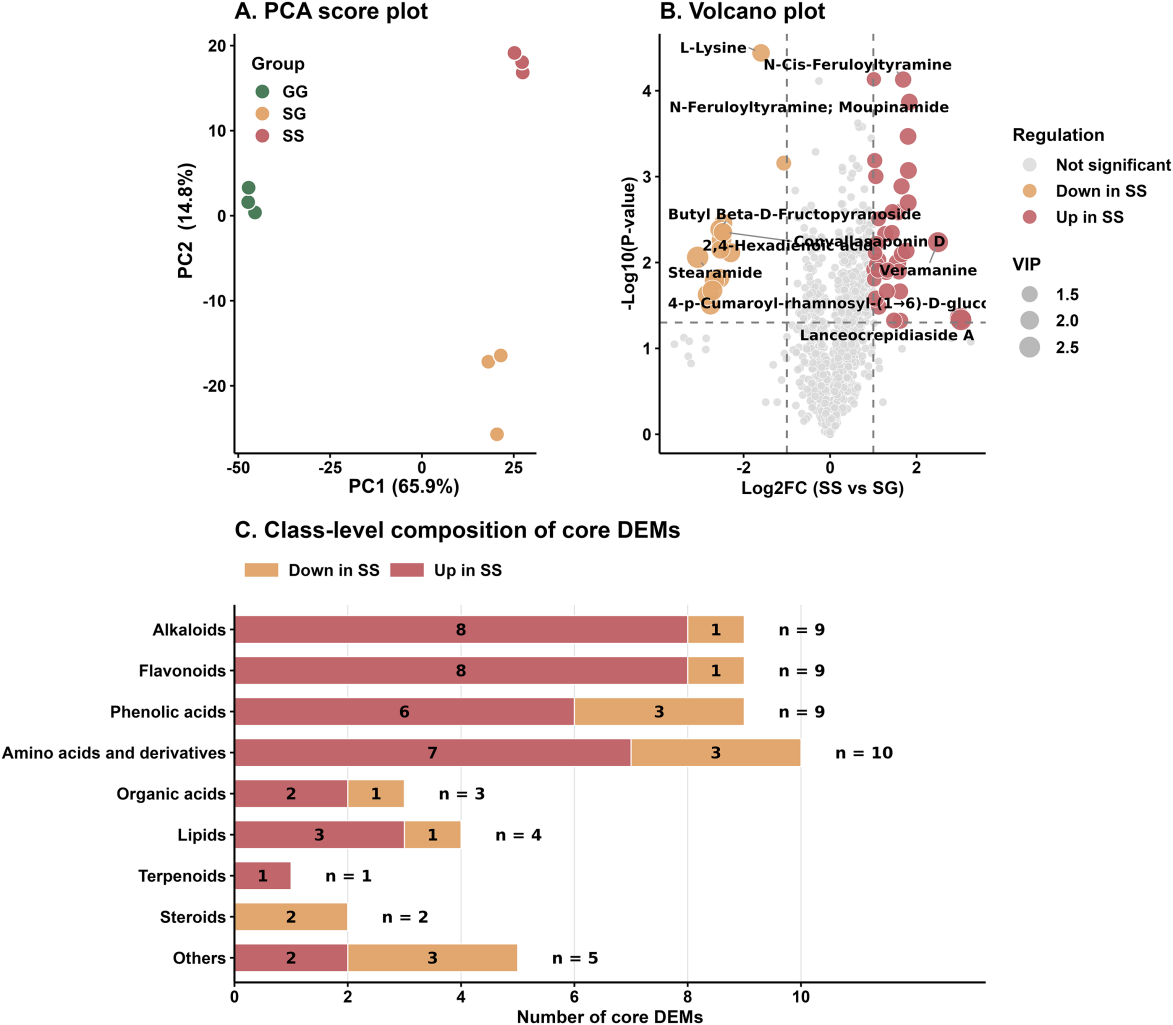
Sector-associated metabolic variation in *A. elatior* leaf sectors. **(A)** Principal component analysis (PCA) score plot of widely targeted LC–MS/MS-based metabolomic profiles across GG, SG, and SS sectors. Each point represents one spatial sector subsample. **(B)** Volcano plot showing differentially accumulated metabolites (DEMs) between SS and SG sectors. Core DEMs were defined using VIP ≥ 1.0, *P* < 0.05, FDR < 0.05, and |log_2_FC| ≥ 1.0. Red and orange points indicate metabolites with higher and lower abundance in SS sectors relative to SG sectors, respectively; grey points indicate non-significant metabolites. **(C)** Class-level composition of the 52 core DEMs. Bars show the numbers of core DEMs assigned to each metabolite class and are separated according to their direction of change in SS relative to SG. Red and orange segments indicate DEMs with higher and lower abundance in SS sectors, respectively. Numbers within bars indicate DEM counts for each direction, and labels on the right indicate the total number of DEMs in each metabolite class.

Widely targeted LC-MS/MS profiling revealed clear metabolic differences among GG, SG, and SS leaf sectors. Principal Component Analysis (PCA) separated the three sector types into distinct clusters (**Figure 4A**), with the first two principal components (PCs) explaining 65.9% and 14.8% of the total variance, respectively. SS samples were clearly separated from both GG and SG in the ordination space, indicating a distinct metabolic profile in the chlorotic sectors.

As SG tissue was collected immediately adjacent to SS, the SS–SG contrast was used to identify metabolites most closely associated with the local chlorotic phenotype. Applying the threshold of VIP ≥ 1.0, *P* < 0.05, FDR < 0.05, and |Log_2_FC| ≥1.0, we identified 52 core differentially accumulated metabolites (DEMs) (**Figure 4B**). Among these, 37 metabolites showed higher abundance in SS tissues, whereas 15 metabolites showed lower abundance.

The 52 core DEMs were then grouped by chemical class and direction of change (**Figure 4C**). They spanned several major categories, among which alkaloids, flavonoids, phenolic acids, and amino acids and derivatives were prominent. Across these classes, metabolites with higher abundance in SS generally outnumbered those with lower abundance, particularly among specialized metabolites. Changes were also evident in amino acid-derived and selected primary metabolism-related compounds, showing that the metabolic differences in SS were not confined to a single biochemical category.

KEGG pathway mapping provided additional functional context for these metabolic differences (**Supplementary Figure 2**). The mapped pathways were mainly associated with broad metabolic and biosynthetic categories, including metabolic pathways (ko01100), biosynthesis of secondary metabolites (ko01110), and biosynthesis of cofactors (ko01240) and biosynthesis of amino acids (ko01230). These assignments were consistent with the observed accumulation of several secondary metabolites and amino acid-derived compounds in SS sectors. By contrast, selected lipid- and primary metabolism-related metabolites showed lower abundance in SS. However, these KEGG results should be interpreted as pathway-level annotations rather than direct evidence of metabolic flux shifts.

Overall, SS sectors displayed a distinct metabolic profile marked by the increased abundance of several alkaloids, flavonoids, and phenolic acids, together with shifts in amino acid-derived and selected primary metabolism-related compounds. These coordinated changes distinguish SS from the adjacent SG tissue and reflect localized metabolic differentiation associated with the chlorotic phenotype.

### 3.6 Transcriptome–metabolome associations linked to localized metabolic variation

To elucidate transcriptional features associated with metabolic differences between SS and SG sectors, an integrated multi-omics analysis between the transcriptome and the widely targeted metabolome was conducted. Pearson correlation coefficients were calculated to construct a correlation-based framework linking differentially expressed genes (DEGs) with the 37 DEMs enriched in SS sectors.

**Figure 5:**
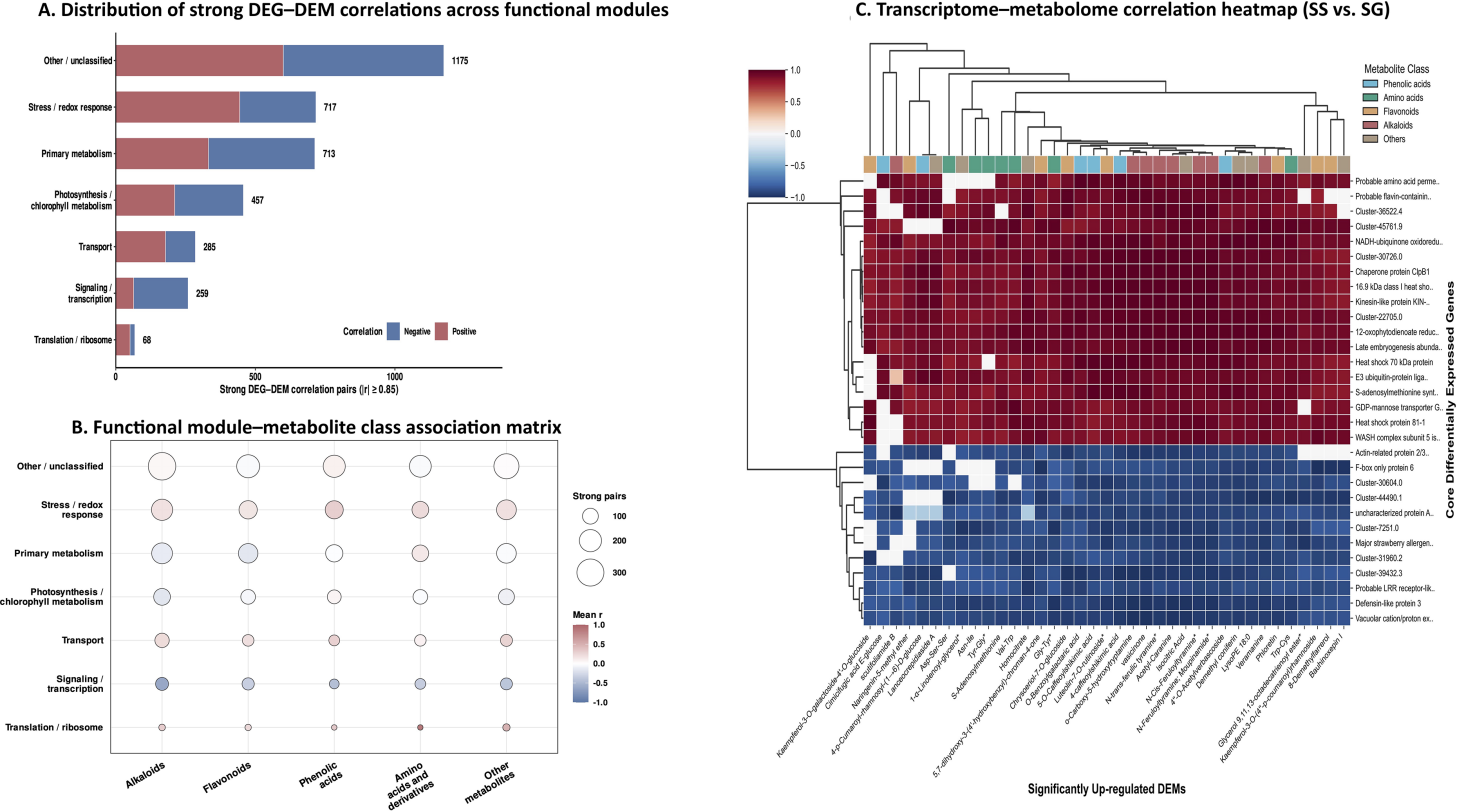
Transcriptome–metabolome association summary between SS and SG sectors. **(A)** Distribution of strong DEG–DEM correlation pairs across gene functional modules (|r| ≥ 0.85, P < 0.05). Bar colors indicate positive and negative correlations, and numbers beside the bars indicate total strong pairs. **(B)** Functional module–metabolite class association matrix. Bubble size represents the number of strong DEG–DEM correlation pairs within each functional module–metabolite class combination, whereas bubble color represents the mean correlation coefficient. The analysis focused on 37 SS-upregulated DEMs. **(C)** Clustered heatmap showing correlations between selected core DEGs and the 37 DEMs with higher abundance in SS relative to SG. Red and blue indicate positive and negative correlation coefficients, respectively, and the annotation bar above the heatmap indicates metabolite class. DEGs, differentially expressed genes; DEMs, differentially accumulated metabolites; SG, adjacent green sector; SS, chlorotic spot sector.

Strong DEG–DEM associations were subsequently assigned to gene functional modules (**Figure 5A**). Using |r| ≥ 0.85 and *P* < 0.05 as the threshold for strong associations, the resulting profile linked the SS-enriched DEMs to genes involved in photosynthesis/chlorophyll metabolism, stress/redox response, primary metabolism, translation/ribosome, transport, and signaling/transcription-related functions. Both positive and negative correlations were observed across these modules, revealing directionally diverse transcriptional patterns associated with the metabolic profile of SS sectors.

The functional module–metabolite class bubble matrix provided a complementary view of these relationships (**Figure 5B**). Bubble size represents the number of strong DEG–DEM correlation pairs within each module–metabolite class combination. Bubble color indicates the corresponding mean correlation coefficient. Associations extended across several classes of metabolites enriched in SS, including alkaloids, flavonoids, phenolic acids, amino acids and derivatives, and other metabolites. At the individual-feature level, the clustered heatmap further resolved correlations between selected DEGs and these SS-enriched DEMs (**Figure 5C**). Collectively, these patterns indicate coordinated variation between gene expression and metabolite accumulation in SS sectors. The observed correlations represent statistical co-variation and should not be interpreted as evidence of direct gene–metabolite regulation.

### 3.7 Associations between endophytic bacterial communities and localized metabolic profiles

**Figure 6:**
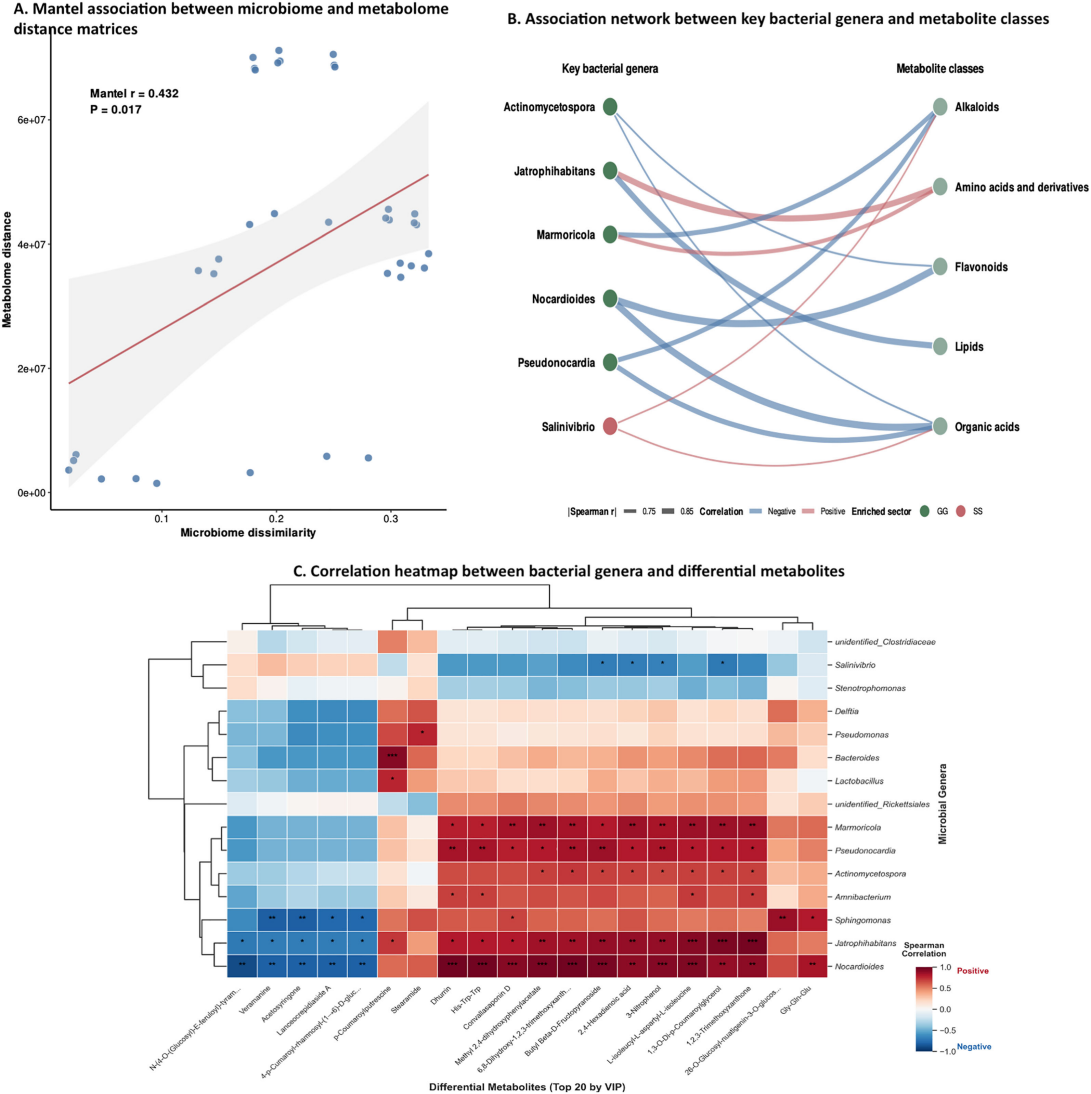
Microbiome–metabolome associations across leaf sectors. **(A)** Mantel test showing the association between microbiome Bray–Curtis dissimilarity and metabolome Euclidean distance based on core DEMs. The Mantel test was performed with 999 permutations, and Mantel r and *P* values are shown in the plot. **(B)** Genus–metabolite class association network showing significant correlations between key bacterial genera and metabolite classes. Edges indicate Spearman correlations with |r| ≥ 0.65 and *P* < 0.05, with the top two strongest associations retained for each genus. Edge color indicates correlation direction, and edge width represents |Spearman r|. GG, normal green sector; SG, adjacent green sector; SS, chlorotic spot sector. **(C)** Hierarchical clustering heatmap showing the Spearman correlations between the 15 most abundant bacterial genera (including 6 LEfSe-identified biomarkers) and the top 20 differential metabolites (based on VIP scores). Both rows and columns are clustered using Ward’s linkage. Color intensity represents the correlation coefficient (red: positive; blue: negative). Asterisks indicate statistical significance: *P < 0.05, **P < 0.01, ***P < 0.001.

We next examined whether sector-associated variation in endophytic bacterial communities coincided with changes in the leaf metabolome. A Mantel test detected a significant positive association between bacterial community structure, represented by Bray–Curtis dissimilarity, and the Euclidean distance matrix derived from the core DEMs (**Figure 6A**; Mantel r = 0.432, *P* = 0.017), indicating that variation in the endophytic bacterial community was associated with variation in the leaf metabolic profile across sectors. At the level of individual features, the correlation heatmap revealed a series of positive and negative associations between representative bacterial genera and differential metabolites (**Figure 6C**), providing a more detailed view of the relationships underlying the overall Mantel association.

These genus–metabolite relationships were further summarized at the metabolite-class level using a bipartite association network (**Figure 6B**). Key bacterial genera identified by LEfSe analysis were linked to major metabolite classes based on significant Spearman correlations. The network condensed the feature-level correlation structure shown in **Figure 6C** and highlighted the principal associations between bacterial taxa and broad metabolite categories.

Several GG-associated bacterial taxa, including *Actinomycetospora*, *Marmoricola*, *Nocardioides*, *Pseudonocardia*, and *Jatrophihabitans*, were associated with multiple metabolite classes, including alkaloids, flavonoids, organic acids, lipids, and amino acids and derivatives. Negative correlations predominated between these GG-enriched genera and alkaloids, flavonoids, and organic acids, consistent with contrasting sector-associated abundance patterns. By contrast, the SS-enriched genus *Salinivibrio* showed positive associations with alkaloids and organic acids, indicating a co-occurrence pattern across SS sectors.

Collectively, the Mantel analysis, feature-level heatmap, and association network reveal a consistent correspondence between endophytic bacterial community composition and localized metabolic variation in *A. elatior* leaves.

### 3.8 Proposed multi-omics association model for SS sectors

Integration of the microbiome, transcriptome, and metabolome datasets yielded a working model of the multi-omics features associated with SS sectors (**Supplementary Figure 3**). This model brings together three complementary layers of evidence: shifts in the endophytic bacterial community, changes in host gene expression, and localized metabolite accumulation.

Within the bacterial community, SS sectors were characterized by enrichment of several bacterial taxa, including *Stenotrophomonas, Salinivibrio*, and unclassified *Rickettsiales*. In contrast, GG sectors were associated with genera such as *Actinomycetospora*, *Nocardioides* and *Jatrophihabitans*. These contrasting distribution patterns suggest sector-associated variation in the relative abundance of endophytic bacterial taxa across leaf tissues. Host transcriptional variation in SS sectors spanned multiple functional modules, including photosynthesis/chlorophyll metabolism, stress/redox response, primary metabolism, translation/ribosome, transport, and signaling/transcription. The involvement of these diverse modules points to broad transcriptional differences encompassing several aspects of host physiology. The metabolic profile of SS sectors was characterized by the localized accumulation of several major metabolite classes, including alkaloids, flavonoids, phenolic acids, amino acids and derivatives, and other metabolites enriched in SS sectors. Together with the transcriptome–metabolome and microbiome–metabolome association analyses, these results reveal coordinated variation among metabolite accumulation, host gene expression, and bacterial community composition in SS tissue.

The resulting model summarizes the principal cross-omics associations observed in chlorotic sectors and provides an integrated view of SS-associated variation in naturally variegated A. elatior leaves.

## 4 Discussion

Leaf variegation in *A. elatior* was accompanied by spatially coordinated differences in endophytic bacterial communities, host gene expression, and metabolism. Although variegated phenotypes have traditionally been interpreted mainly in terms of host genetics, physiological defects, or viral pathogens, our multi-omics data provide an additional microbiome-associated perspective on this localized phenotypic trait. We observed sector-associated variation in bacterial taxonomic composition and host specialized metabolism across GG, SG, and SS sectors. These results are consistent with phyllosphere and endosphere studies showing that leaf-associated microbiomes are responsive to host developmental status, environmental factors, and local metabolic conditions (Cui et al., 2024; Geyer et al., 2024; Tan et al., 2025; Feng and Liu, 2025). In addition, experimental manipulation of phyllosphere bacterial communities has been shown to alter plant microbiome composition and leaf traits, supporting a potential link between microbial community variation and host phenotypic changes (Ohler et al., 2022).

### 4.1 Sector-associated bacterial community shifts and host transcriptomic responses

SS sectors were enriched in unclassified *Rickettsiales*, *Stenotrophomonas*, and *Salinivibrio*, several of which have previously been detected in plant-associated communities under stressful conditions. *Stenotrophomonas species*, for example, have been reported to colonize stressed host environments and display diverse resource-scavenging capacities (Wang et al., 2025, 2025). By contrast, several *Actinobacteriota*-associated taxa were less abundant in SS sectors. Members of this phylum have been implicated in maintaining phyllosphere or endosphere stability in diverse host systems (Chen et al., 2020; Schäfer et al., 2023; Meng et al., 2025; Ehau-Taumaunu et al., 2025).

Here, bacterial community imbalance is used as an operational term to describe the SS-associated pattern of lower Shannon diversity relative to SG sectors, pronounced taxonomic dominance, and depletion of GG-associated *Actinobacteriota* taxa. This term does not imply a confirmed disease state or establish bacterial imbalance as the primary cause of leaf variegation. Thus, the contrast between SS-associated taxa and GG-associated *Actinobacteriota* taxa may reflect localized variation in bacterial taxonomic composition across leaf sectors rather than a discrete pathological state.

Our integrated analyses further suggested that SS-associated bacterial community variation co-occurred with host stress- or defense-related transcriptional features and localized metabolic changes. Plant immune and stress-response pathways are known to participate in host–microbiome interactions and can shape microbial colonization patterns in leaf-associated niches (Chaudhry et al., 2021; Sorger et al., 2026). Among the genes with high connectivity in the correlation network were those encoding heat shock protein 70 (HSP70) and the ubiquitination-related F-box protein SKIP23. Their prominence is consistent with a transcriptional state involving cellular stress responses, protein homeostasis, and immune-related signaling (Zhang et al., 2019; Zhao et al., 2024). This broader host response provides a physiological context for the sector-associated metabolic patterns examined in the following section.

### 4.2 Sector-associated metabolic variation and stress-related specialized metabolites

The transcriptomic features observed in SS tissues were accompanied by a distinct metabolic profile, with 37 metabolites showing higher abundance in SS than in the adjacent SG tissue. These compounds were distributed mainly among alkaloids, flavonoids, phenolic acids, and amino acid derivatives, indicating that the chlorotic sectors differed not only in primary physiological status but also in the accumulation of specialized metabolites. In parallel, the transcriptome–metabolome association analysis showed that functional gene modules related to primary metabolism, transport, and stress/redox response were associated with multiple SS-accumulated metabolite classes, indicating coordinated variation between transcriptional activity and metabolic accumulation in SS sectors.

Specialized metabolites can influence microbial community assembly and may participate in host–microbiome interactions under stress conditions (Chao et al., 2025; Wang et al., 2025). Flavonoids and phenolic acids can contribute to antioxidant defense and the buffering of cellular oxidative stress (Mostafavi Abdolmaleky and Zhou, 2024; Hou et al., 2025), whereas altered redox conditions may influence the stability of host-associated microbial communities (Wang et al., 2019; da Cruz Nizer et al., 2024). This interpretation is further supported by stable-isotope-assisted metabolomic evidence showing that aromatic amino acid metabolism can supply a range of defense-related specialized metabolites, including hydroxycinnamic acid derivatives, glycosides, quinates, and lignans, during biotic stress (Rypar et al., 2025). Thus, the accumulation of phenolic acids, flavonoids, and amino acid-derived metabolites in SS tissues is consistent with a localized stress-responsive metabolic state. These metabolic features also provide a chemical context for the sector-associated bacterial and transcriptional patterns described above.

### 4.3 Interpretation of the integrated microbiome–host association model

Unlike stress scenarios in which both bacterial and fungal communities may respond to host or environmental perturbations (Ehau-Taumaunu et al., 2025; Yang et al., 2025), the fungal community recovered from surface-sterilized leaf tissues showed comparatively stable compositional patterns across GG, SG, and SS sectors in the present dataset. Genera such as *Fusarium* and *Aspergillus* were detected across all sector types, suggesting that no clear sector-restricted fungal compositional shift was resolved. However, this apparent stability does not preclude fungal involvement, because ecologically relevant differences may occur at the strain or functional level, through cross-kingdom interactions, or among low-abundance taxa that are poorly resolved by amplicon sequencing (del Barrio-Duque et al., 2019, 2020; Maggini et al., 2023; Ali et al., 2024).

The proposed multi-omics model should therefore be interpreted as an association-level framework rather than as a linear mechanistic pathway. Although SS sectors showed coordinated variation in bacterial community structure, host transcriptomic responses, and metabolite accumulation, the current data do not establish whether bacterial shifts precede, result from, or occur in parallel with host physiological changes. Such directionality is difficult to infer because plant-associated microorganisms respond to host metabolic conditions while also having the capacity to modify host stress physiology (Lei et al., 2023; Zhang et al., 2024). Alternatively, several non-exclusive scenarios may explain the observed associations. For instance, bacterial community imbalance may contribute to localized host responses and metabolic variation in SS sectors. Sector-specific host physiological changes and specialized metabolite accumulation may reshape the local microenvironment and favor particular bacterial taxa (Sahil et al., 2026). Reciprocal feedback between host sector phenotype and endophytic bacterial community structure may reinforce localized microbiome–metabolome patterns (Singh et al., 2022; Wang et al., 2024). These possibilities cannot be resolved from cross-sectional correlation analyses alone.

### 4.4 Ecological implications and future perspectives

Our findings provide a microbiome-associated perspective for understanding localized leaf variegation in *A. elatior*. Rather than viewing chlorotic sectors solely as passive manifestations of pigment loss or cellular dysfunction, our results indicate that SS sectors represent localized microenvironments associated with variation in bacterial taxonomic composition, host transcriptional responses, and specialized metabolite accumulation. The accumulation of alkaloids, flavonoids, and phenolic acids in SS tissues, together with stress-related transcriptional features, suggests that chlorotic sectors may involve localized stress-associated metabolic adjustment within spatially defined leaf regions. These observations support the value of a spatially resolved multi-omics framework for investigating plant–microbiome–metabolite associations in variegated leaves, while remaining within an association-level interpretation.

The three-sector metabolomic analysis distinguished SS from both GG and SG, whereas the subsequent differential and transcriptome–metabolome analyses focused on the SS–SG contrast. As SG was sampled adjacent to SS, this comparison provided the most direct spatial reference for resolving molecular variation across the local chlorotic–green transition. Extending the same analytical framework to SS and GG would help determine whether these associations also characterize the broader contrast between chlorotic and normal green tissues. The sector-level sampling design captures within-leaf spatial variation but does not allow inference of causality or temporal dynamics. Future studies using independent biological replicates, time-series sampling, and functional validation (*e.g*., microbial isolation and inoculation assays) will be required to test the robustness of these associations.

## 5 Conclusions

Integrated profiling of the microbiome, transcriptome, and metabolome revealed marked molecular and microbial heterogeneity among adjacent leaf sectors of *A. elatior*. SS sectors were characterized by sector-associated bacterial taxonomic patterns and lower Shannon diversity relative to SG, whereas fungal community composition remained comparatively stable across sectors. These bacterial differences coincided with host transcriptional features related to stress and defense, together with increased accumulation of several specialized metabolites, particularly alkaloids, flavonoids, and phenolic acids. By resolving these changes within individual leaves, our study places localized variegation in a broader microbiome–host–metabolite context and highlights chlorotic sectors as spatially differentiated biological microenvironments.

## Data Availability

The datasets presented in this study can be found in online repositories. The names of the repositories and accession numbers can be found below: (1) The 16S rRNA, ITS amplicon sequencing, and transcriptomic raw data are available in the NCBI Sequence Read Archive (SRA) under BioProject accession number(s) PRJNA1450849. (2) The widely targeted metabolomics data presented in this study are available on request from the corresponding author.

## Supplementary material

Supplementary Figure 1 Rarefaction curves for bacterial 16S amplicon sequencing. Rarefaction curves were generated based on bacterial ASV profiles after computational removal of host chloroplast and mitochondrial sequences.

Supplementary Figure 2 KEGG pathway summary of annotated differential metabolites. The top 10 KEGG pathways are shown according to the number of mapped significant compounds. Bubble size represents the number of significant compounds mapped to each pathway, and bubble color indicates the proportion of mapped significant compounds among all compounds assigned to the pathway. Significant compounds were defined as DEMs between SS and SG sectors using VIP ≥ 1.0, *P* < 0.05, FDR < 0.05, and |log_2_FC| ≥ 1.0.

Supplementary Figure 3 Proposed association model summarizing multi-omics patterns in chlorotic spot sectors of naturally variegated *A. elatior* leaves. The model integrates sector-associated changes in the endophytic bacterial community, host transcriptome, and metabolome. SS sectors were associated with enrichment of several bacterial taxa, host transcriptomic responses related to photosynthesis/chlorophyll metabolism, stress/redox response, primary metabolism, translation/ribosome, and signaling/transport, and localized accumulation of major metabolite classes, including alkaloids, flavonoids, phenolic acids, and amino acid derivatives. Red arrows indicate increased or SS-associated features, green arrows indicate GG-associated or relatively reduced features in SS sectors, and grey arrows indicate association-based links between omics layers. This model represents an association-based working hypothesis and does not imply direct causality.
