# Supplementary figures and images for: Multi-omics analysis reveals associations among endophytic microbiome shifts, host transcriptional responses, and metabolic variation across variegated leaf sectors of Aspidistra elatior
Source: Front Plant Sci. 2026 Jul 8;17:1860906. doi: 10.3389/fpls.2026.1860906 (PMC13388911; doi:10.3389/fpls.2026.1860906)

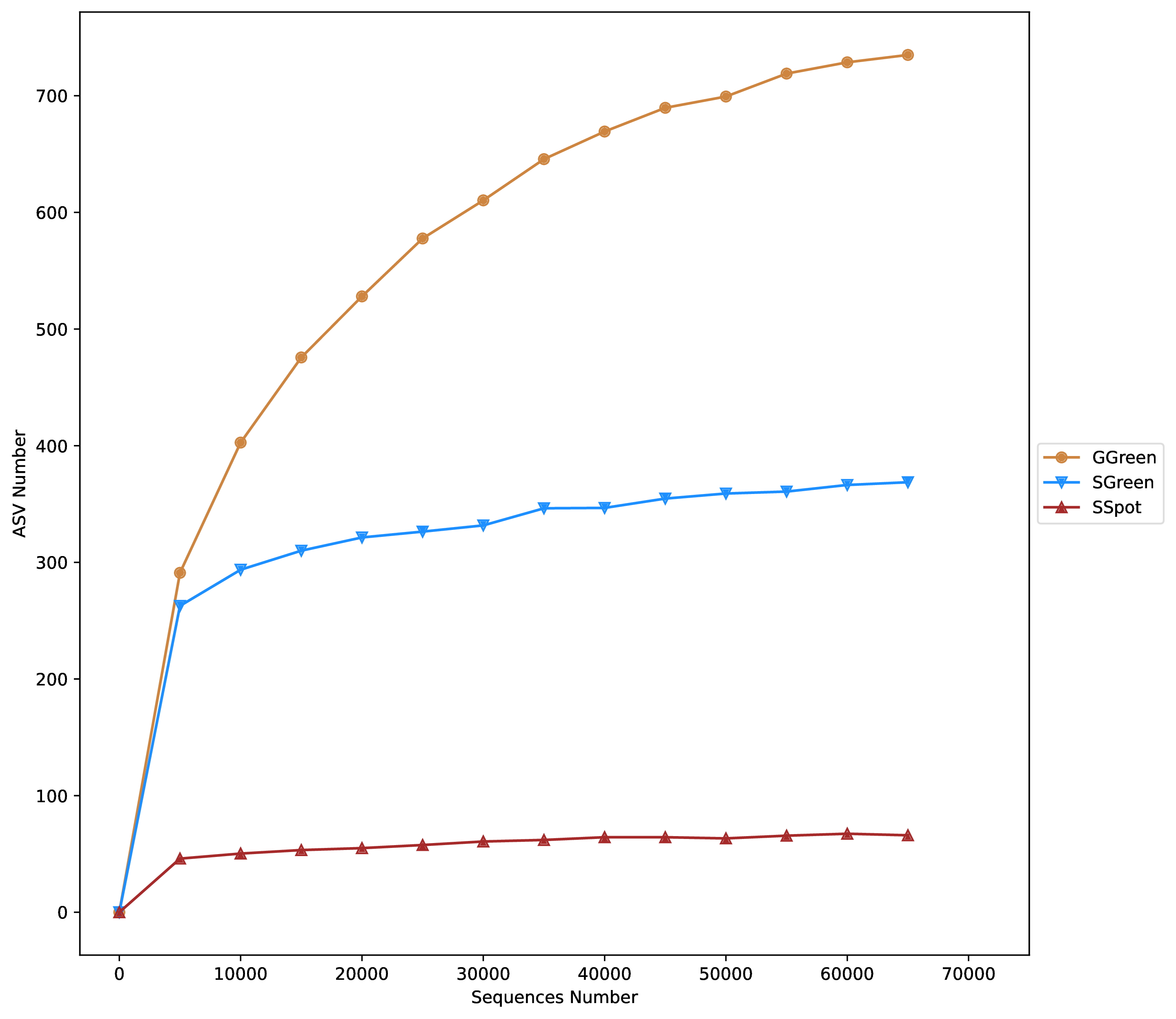

Supplement: Supplementary Figure 1 — Rarefaction curves for bacterial 16S amplicon sequencing. Rarefaction curves were generated based on bacterial ASV profiles after computational removal of host chloroplast and mitochondrial sequences. [file Image1.jpeg]

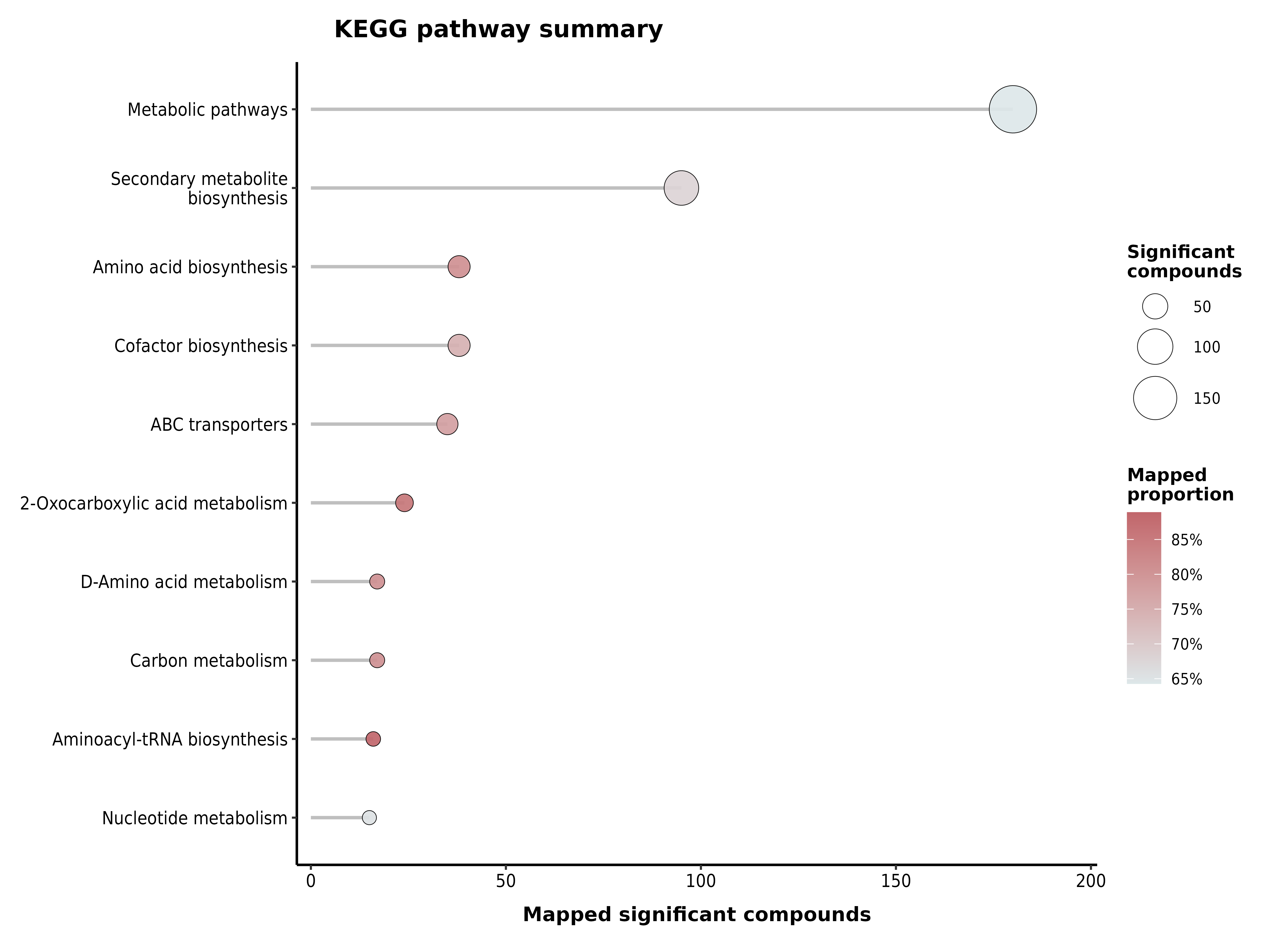

Supplement: Supplementary Figure 2 — KEGG pathway summary of annotated differential metabolites. The top 10 KEGG pathways are shown according to the number of mapped significant compounds. Bubble size represents the number of significant compounds mapped to each pathway, and bubble color indicates the proportion of mapped significant compounds among all compounds assigned to the pathway. Significant compounds were defined as DEMs between SS and SG sectors using VIP ≥ 1.0, P < 0.05, FDR < 0.05, and |log2FC| ≥ 1.0. [file Image2.tiff]

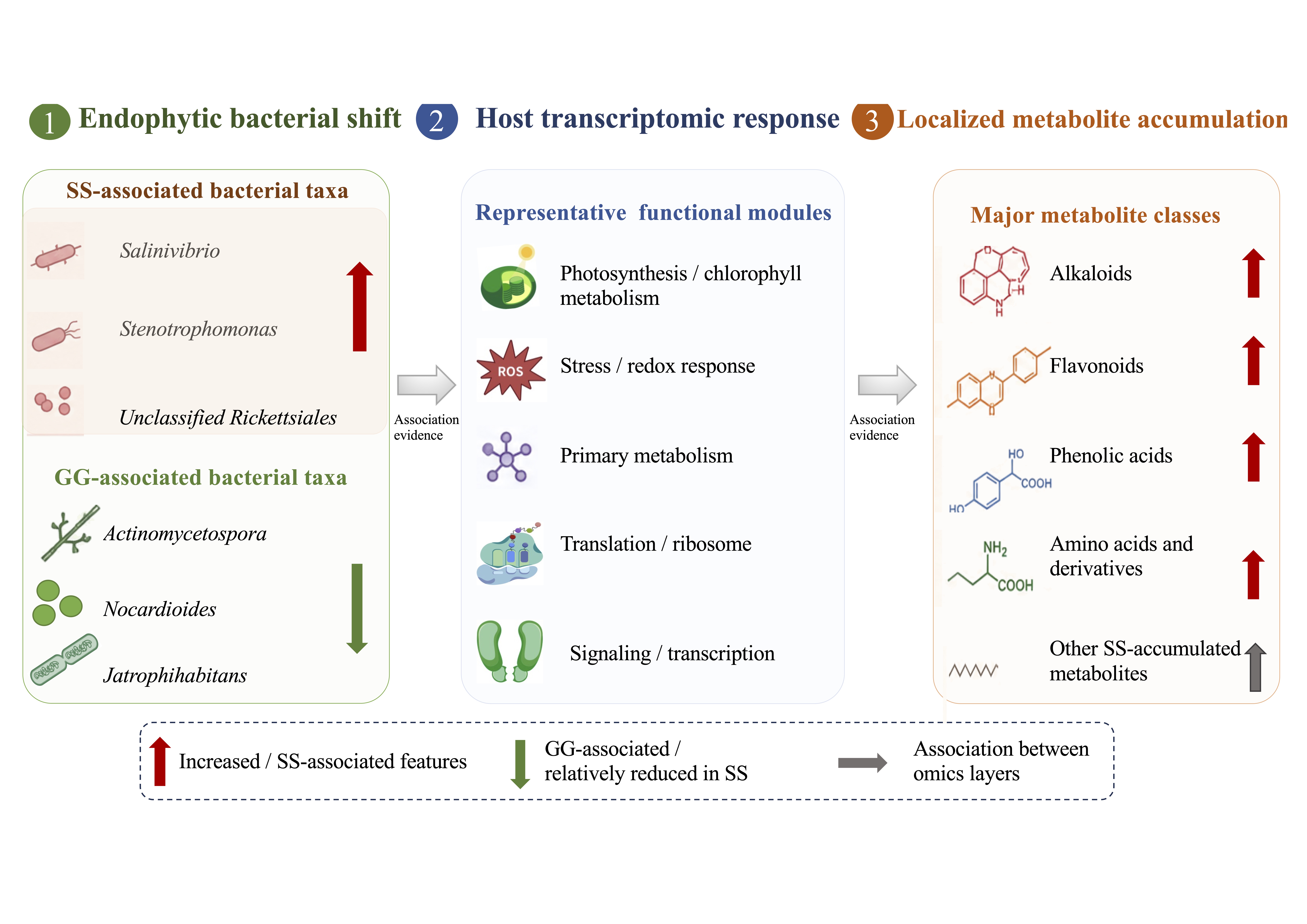

Supplement: Supplementary Figure 3 — Proposed association model summarizing multi-omics patterns in chlorotic spot sectors of naturally variegated A. elatior leaves. The model integrates sector-associated changes in the endophytic bacterial community, host transcriptome, and metabolome. SS sectors were associated with enrichment of several bacterial taxa, host transcriptomic responses related to photosynthesis/chlorophyll metabolism, stress/redox response, primary metabolism, translation/ribosome, and signaling/transport, and localized accumulation of major metabolite classes, including alkaloids, flavonoids, phenolic acids, and amino acid derivatives. Red arrows indicate increased or SS-associated features, green arrows indicate GG-associated or relatively reduced features in SS sectors, and grey arrows indicate association-based links between omics layers. This model represents an association-based working hypothesis and does not imply direct causality. [file Image3.jpeg]
